# Scalable Production of 2D Material Heterostructure Textiles for High-Performance Wearable Supercapacitors

**DOI:** 10.1021/acsnano.3c06181

**Published:** 2023-09-11

**Authors:** Md Rashedul Islam, Shaila Afroj, Nazmul Karim

**Affiliations:** † Centre for Print Research (CFPR), University of the West of England (UWE), Frenchay Campus, Bristol BS16 1QY, U.K.; ‡ National Graphene Institute (NGI), University of Manchester, Oxford Road, Manchester M13 9PL, U.K.; § Advanced Textiles Research Group, Nottingham Trent University, Shakespeare Street, Nottingham NG1 4GG, U.K.

**Keywords:** graphene, 2D materials, heterostructure, wearable electronics, e-textiles, supercapacitors

## Abstract

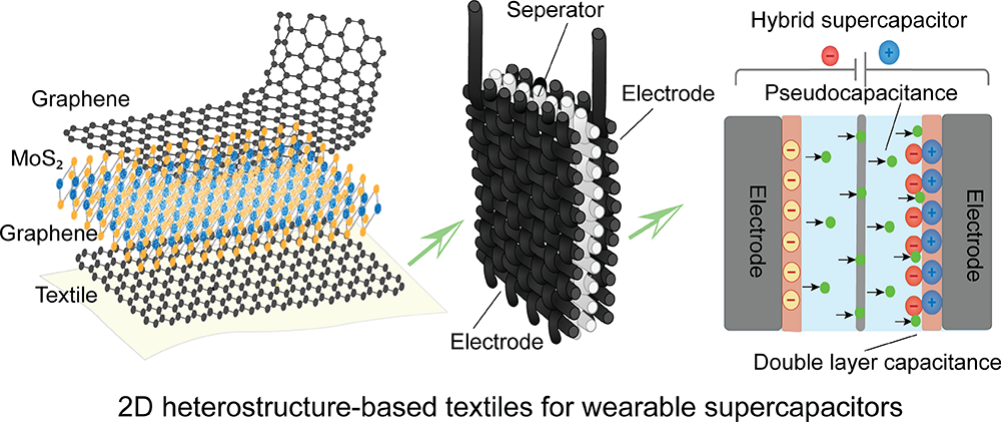

Wearable electronic textiles (e-textiles) have emerged as a promising platform for seamless integration of electronic devices into everyday life, enabling nonintrusive monitoring of human health. However, the development of efficient, flexible, and scalable energy storage solutions remains a significant challenge for powering such devices. Here, we address this challenge by leveraging the distinct properties of two-dimensional (2D) material based heterostructures to enhance the performance of wearable textile supercapacitors. We report a highly scalable and controllable synthesis method for graphene and molybdenum disulfide (MoS_2_) through a microfluidization technique. Subsequently, we employ an ultrafast and industry-scale hierarchical deposition approach using a pad-dry method to fabricate 2D heterostructure based textiles with various configurations suitable for wearable e-textiles applications. Comparative analyses reveal the superior performance of wearable textile supercapacitors based on 2D material heterostructures, demonstrating excellent areal capacitance (∼105.08 mF cm^–2^), high power density (∼1604.274 μW cm^–2^) and energy density (∼58.377 μWh cm^–2^), and outstanding capacitive retention (∼100% after 1000 cycles). Our findings highlight the pivotal role of 2D material based heterostructures in addressing the challenges of performance and scalability in wearable energy storage devices, facilitating large-scale production of high-performance wearable supercapacitors.

Wearable electronics have revolutionized the field of personalized healthcare by enabling nonintrusive monitoring of human health during daily life.^1^ However, the full-scale deployment of wearable electronic textiles, commonly known as e-textiles, faces significant challenges in terms of powering these devices while maintaining essential textile properties such as flexibility, durability, lightweight, biocompatibility, and strength.^2^ The increasing demand for wearable electronic devices and the need for efficient energy storage systems have spurred significant advancements in the field of textile-based wearable supercapacitors.^3^ These devices, integrated seamlessly into fabrics, hold immense potential for powering wearable electronics, healthcare monitoring systems, and smart textiles.^4−7^ However, scalable production of high-performance supercapacitors that combine excellent electrochemical properties with the flexibility and comfort of textiles remains a considerable challenge.^8^ To overcome these , there is a pressing need to explore advanced materials and innovative design approaches that can enhance the energy storage performance of wearable textile supercapacitors.

Two-dimensional (2D) material heterostructures offer a compelling solution to enhance energy performance by combining different ingredients into a single ultimate structure.^9^ These heterostructures, formed by stacking 2D materials with complementary properties, exhibit enhanced properties that are not found in the individual materials. By carefully arranging the layers, researchers can manipulate interlayer interactions and band structures, resulting in the creation of diverse tailor-made heterostructures with specific and tailored properties.^10,11^ Electrochemical capacitors store electrical energy either in the electrochemical double layer (EDL) formed by electrolyte ions on the electrode surface or through redox reactions involving the electrode material’s surface regions, known as pseudocapacitance.^12,13^ However, materials that possess both of these properties are rare but crucial for robust and efficient devices.^14^ To address this, the fabrication of 2D material based heterostructures combining EDLC and pseudocapacitive materials becomes essential, as it increases the surface area and enhances the active electrochemical sites in the superior heterostructure. This architecture enables energy storage through near-surface ion adsorption and additional contribution from fast reversible Faradaic reactions, leading to high energy and power density.^15,16^ Therefore, 2D material heterostructures offer a versatile approach to enhance energy storage performance through their distinct properties, enabling the development of highly efficient and high-capacity electrochemical capacitors.

The isolation of graphene in 2004^17^ unveiled a diverse range of graphene-like 2D materials (2DM) with exceptional mechanical, thermal, and electrical properties.^18−20^ These materials enable the creation of heterostructures with diverse properties. In our previous works, we demonstrated the potential of graphene and its derivatives as functional materials for next-generation wearable electronic textiles.^21−25^ Additionally, we utilized graphene as an efficient supercapacitor electrode material for powering wearable electronic devices.^26,27^ Transition-metal dichalcogenides (TMDs) have attracted significant attention due to their distinct physical properties such as magnetism, charge-density-wave order, superconductivity, and potential applications in high-performance electronic devices.^28^ TMDs exhibit improved energy storage capabilities compared to traditional electrode materials and other 2D materials like graphene, thanks to their layered structures with sufficient interlayer space.^29^ Among the various TMD materials, 2D MoS_2_ exhibit good capacitive properties,^30^ due to the tunable band gap and a large number of active sites with extraordinary physical and chemical properties.^31^ Despite their favorable properties, MoS_2_ has limitations, including restacking, unsatisfactory electrical conductivity, inflexibility, and poor interface quality in electronic and electrochemical devices. To overcome these challenges, researchers have explored a wide variety of MoS_2_-based heterostructures. Although graphene-MoS_2_ heterostructures have been reported for other applications including nonvolatile memory cells,^32^ superlattice configuration,^33^ fiber lasers,^34^ and supercapacitor electrodes on a nickel^35^ or graphite^36^ substrate, their utilization in textile-based wearable supercapacitors remains unexplored.

To fully realize the potential of 2D material based wearable textile supercapacitors, several challenges pertaining to performance and scalable manufacturing must be addressed. These challenges encompass the achievement of high energy storage performance while preserving the desired textile properties of flexibility, durability, light weight, and biocompatibility. Additionally, the development of scalable production methods for synthesizing and integrating 2D materials into textile structures is pivotal for enabling large-scale manufacturing of high-performance wearable supercapacitors. To address these challenges, here we report a scalable microfluidization technique for the production of two prominent 2D materials: graphene and MoS_2_. Leveraging the advantages of a microfluidization technique, we successfully exfoliate graphene and MoS_2_ dispersions and subsequently hierarchically deposit them in various heterostructure configurations on textiles by using a highly scalable and ultrafast pad-dry method. By harnessing the exceptional properties of 2D material based heterostructures, we demonstrate the potential to significantly enhance the energy storage performance of wearable textile supercapacitors while ensuring scalability in manufacturing.

## Results and Discussion

### System Overview

**Figure 1:**
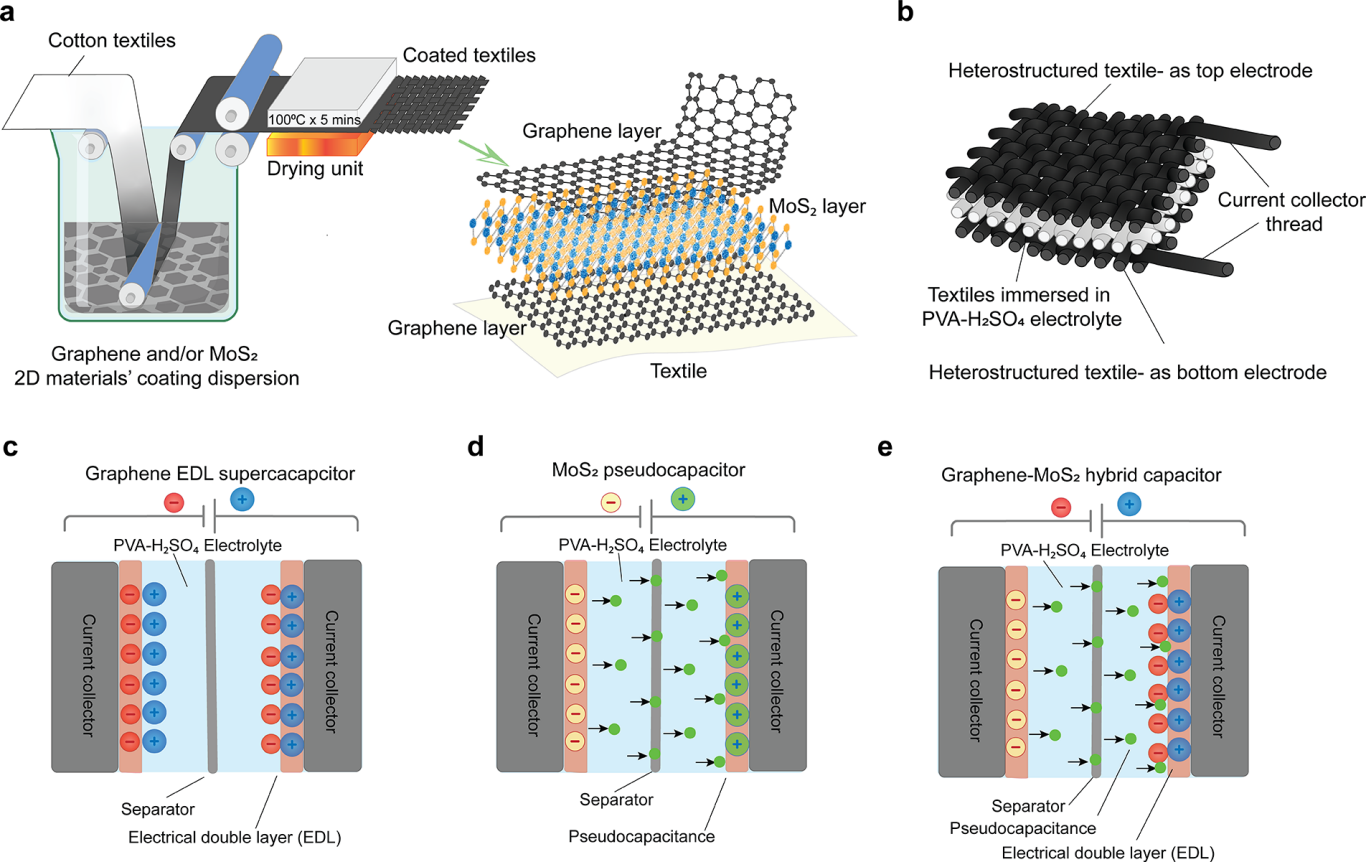
System overview of the 2D material heterostructure textile for supercapacitor applications (a) Scalable pad-dry method for coating of graphene and/or MoS_2_ materials for textiles. (b) Schematic of the supercapacitor structure based on heterostructure textiles electrode (c) Graphene based electrical double layer (EDL) supercapacitor. (d) MoS_2_-based pseudocapacitor. (e) Graphene-MoS_2_-graphene heterostructure based hybrid supercapacitor.

2D material heterostructures offer a platform for tailoring and combining the distinct properties of different 2D materials, enabling the discovery of phenomena and the development of advanced devices and technologies across multiple disciplines.^37^ In this study, we report graphene and MoS_2_ heterostructures_,_ a specific combination of two different 2D materials, stacked together in a layered structure on textile fabrics (Figure 1). The graphene layer can provide higher electrical conductivity, while the MoS_2_ layer contributes to the tunable band gap. In such a heterostructure, the band alignment can be modified by changing the stacking configuration or introducing strain. This tunability offers control over the charge transfer, carrier dynamics, and optical properties of the heterostructure, enabling the design of devices with tailored electronic characteristics and efficient charge transport. A highly scalable pad-dry method (Figure 1a) was used to stack graphene and MoS_2_ in different configurations on textiles. The heterostructure textiles (Figure 1b) were then utilized as supercapacitor electrodes. While graphene-based supercapacitors work as EDL capacitors (Figure 1c) and MoS_2_-based supercapacitors work as pseudocapacitors (Figure 1d), the 2D heterostructure based textile supercapacitors work as hybrid capacitors (Figure 1e). We initially varied the number of graphene coating layers for graphene based and number of MoS_2_ coating layers for MoS_2_ based textile supercapacitors. We then attempted to explore supercapacitor performance fabricated from the MoS_2_-graphene bilayer coated electrodes and graphene-MoS_2_-graphene trilayer coated textile electrodes (Table S1, Section 1 in the Supporting Information).

### Scalable Production of 2D Materials via Microfluidization Technique

We use a highly scalable microfluidization technique to exfoliate few-layer two-dimensional (2D) materials (graphene and MoS_2_ flakes) from graphite and MoS_2_ in water-based dispersions. A microfluidizer generates liquid velocities of 400 ms^–1^ and several orders of magnitude higher shear rates (>10^8^ s^–1^) than conventional rotor-based or other homogenizers by passing fluids through microchannels (diameter, *d* < 100 μm) at high pressure (up to 209 MPa).^38^ Though primarily used for particle size reduction,^39^ nanoemulsion of immiscible liquids,^40^ disrupting or lysing cells,^41,42^ and deagglomeration and dispersion of carbon nanotubes (CNTs) and graphene nanoplatelets (GNP) into polymers,^43^ few studies have highlighted the use of the microfluidization technique to produce graphene,^38,44^ graphene quantum dots,^37^ and two-dimensional (2D) boron nitride nanosheets.^45^ It is also a simple and environmentally friendly technique with 100% exfoliation yield.^44^

**Figure 2:**
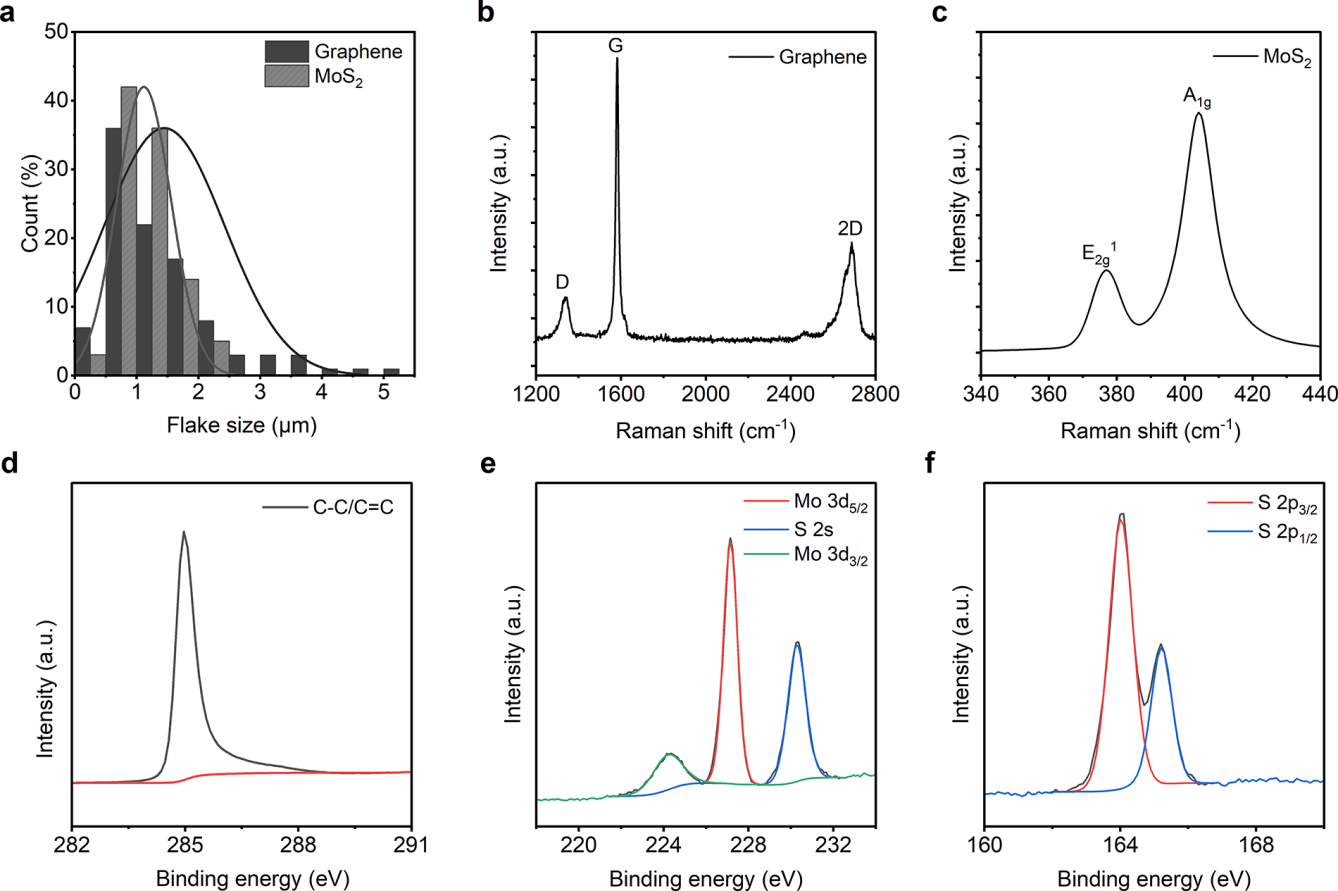
Characterization of microfluidized 2D materials. (a) Size distribution of graphene and MoS_2_ flakes. (b) Raman spectrum of graphene flakes. (c) Raman spectrum of MoS_2_ flakes. (d) High-resolution XPS spectra of graphene flakes. High-resolution XPS spectra of (e) Mo 3d and S 2s and (f) S 2p of as-prepared MoS_2_ flakes.

Figure 2a shows that the average lateral size of exfoliated graphene flakes is ∼1.45 μm and that of the MoS_2_ flakes is ∼1.25 μm. Figure 2b shows a Raman spectrum of exfoliated graphene flakes after 20 cycles, a typical spectrum for liquid-phase exfoliated graphene, with the characteristic D peak at ∼1350 cm^–1^, G peak at ∼1582 cm^–1^ and an asymmetric 2D band at ∼2730 cm^–1^.^38,44^Figure 2c shows the Raman spectrum of exfoliated MoS_2_ flakes. Similar to graphene, single-layer and few-layer MoS_2_ have distinctive signatures in the Raman spectrum. It contains two prominent peaks: an in-plane (E_2g_) mode located around ∼386 cm^–1^ and an out-of-plane (A_1g_) mode, which is located at ∼404 cm^–1^. The in-plane mode corresponds to the sulfur atoms vibrating in one direction and the molybdenum atom in the other, while the out-of-plane mode is a mode of just the sulfur atoms vibrating out-of-plane. The difference between these two modes (∼18 cm^–1^) can be used as a reliable identification for monolayer MoS_2_.

X-ray photoelectron spectroscopy (XPS) measurements were performed to investigate the chemical composition and phase state of the as-prepared graphene and MoS_2_ flakes. Figure 2d shows the high-resolution C 1s spectra, which reveals peaks for graphene flakes, dominated by C–C and C–C=C in aromatic rings (∼284.6 eV). The high-resolution XPS spectrum of the exfoliated MoS_2_ acquired in the chalcogen binding energy region (226–232 eV)^46^ exhibits two obvious peaks at ∼227 and ∼230.5 eV, corresponding to Mo 3d_5/2_ and Mo 3d_3/2_, respectively, indicating the characteristic of a Mo^4+^ state in MoS_2_^47^ (Figure 2e). The peaks of S 2p_3/2_ and S 2p_1/2_ were located at ∼164.0 and ∼165.1 eV, respectively, with a spin–orbit splitting of 1.1 eV, revealing the S_2_^–^ in MoS_2_^48,49^ (Figure 2f).

### Scalable Production of Highly Flexible Graphene-MoS_2_ Heterostructure-Based E-textiles

In the textile industry, the pad-dry method is widely employed for applying functional finishes to textiles, such as antimicrobial, water repellency, wrinkle resistance, and moisture management finishes. This method offers a higher production speed, capable of processing ∼150 m fabrics with functional finishes in just 1 min. In our study, we utilized a laboratory-scale pad-drying unit designed to mimic the industrial counterpart, demonstrating its potential for large-scale production of e-textiles. To simulate the industrial process, we passed a white cotton control fabric, which had undergone desizing, scouring, and bleaching processes to remove impurities and colors, through a padding bath containing a 10 g L^–1^ graphene dispersion. The mangle’s nip rollers were used to remove excessive dispersions from the fabric’s surface, ensuring uniform coating. As a result of the black color of the graphene dispersion, the white fabric quickly transformed into black shortly after coating, typically within seconds. Subsequently, the coated fabric was dried at 100 °C for 5 min in a laboratory dryer to eliminate water/solvent and fix the graphene onto textiles. We repeated this process for up to 10 successive coating layers on graphene-based textiles.

**Figure 3:**
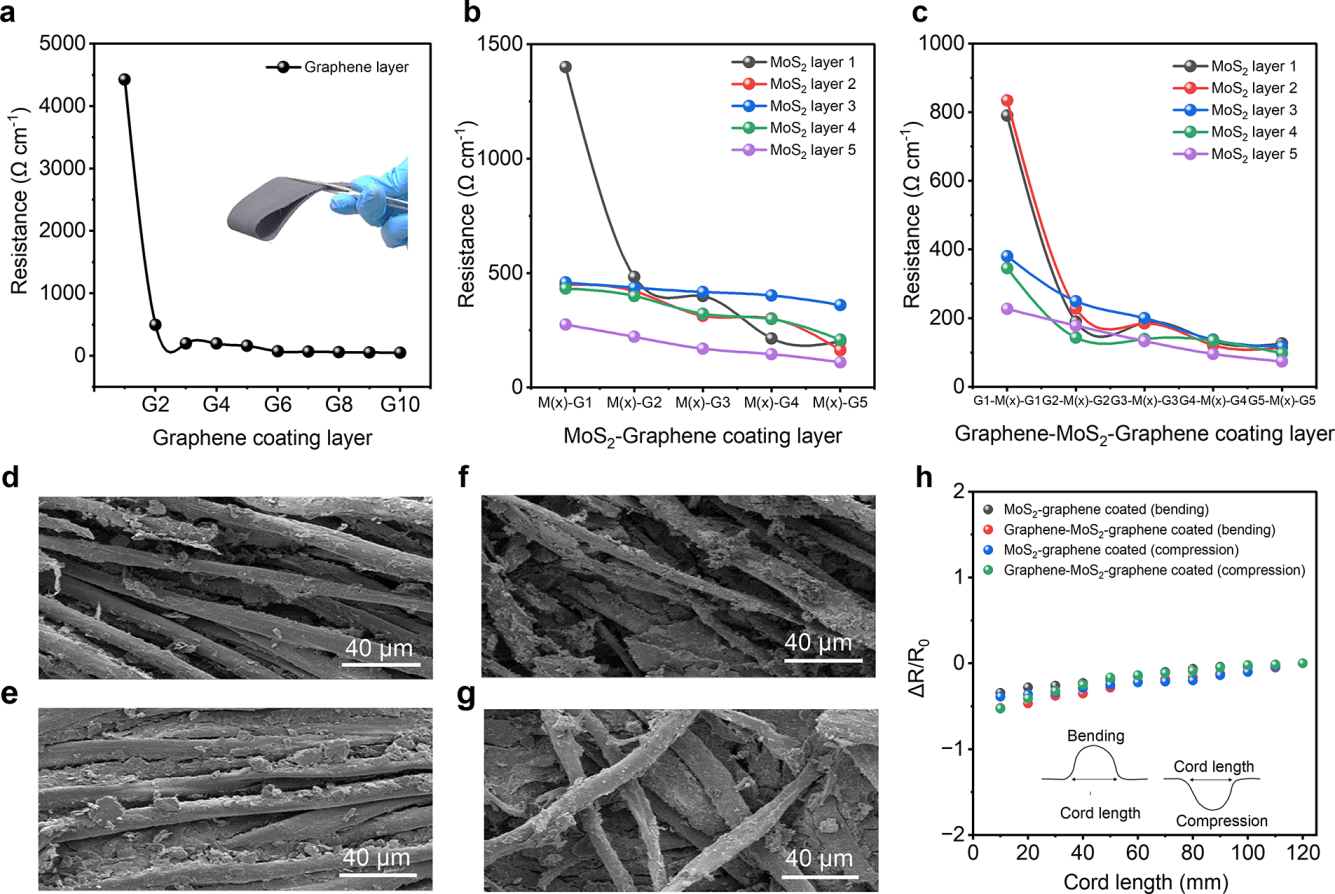
Characterization of the 2DM heterostructure based textiles. The change in the electrical resistance of (a) graphene coated, (b) MoS_2_-graphene bilayer coated, and (c) graphene-MoS_2_-graphene trilayer coated textiles. Surface morphology of the 2DM coated textiles: scanning electron microscope (SEM) image of the (d) graphene 1 coated textiles, G1 (×1000), and (e) graphene 10 coated textiles, G10 (×1000). (f) 4 MoS_2_-5 graphene coated textiles, M4G5 (×1000) and (g) 4 graphene-3 MoS_2_-4 graphene coated textiles, G4M3G4 (×1000). (h) Variation in resistance of MoS_2_-graphene bilayer coated and graphene-MoS_2_-graphene trilayer coated textiles during bending and compression.

Figure 3a shows the changes in electrical resistance per unit length of the graphene-coated fabric with the number of coating layers. Following the application of a single coating layer with graphene dispersion, the resistance of the fabric was measured to be ∼4426.5 Ω cm^–1^. Notably, the resistance underwent a significant reduction of ∼89% after the second coating layer, reaching ∼493.5 Ω cm^–1^. Furthermore, the resistance continued to decrease with each additional coating layer, reaching ∼71.33 Ω cm^–1^ after six layers. We continued the coating process up to ten layers, resulting in a minimum resistance of ∼49.62 Ω cm^–1^. This observed phenomenon can be attributed to absorption and adsorption mechanisms. Initially, the graphene dispersion is absorbed into the textile fibers, leading to a significant reduction in the resistance during the first few coating cycles. As saturation is reached, the dispersion predominantly adsorbs onto the surface of the textiles, forming a continuous conductive film by establishing improved connections between graphene flakes. As evidenced from the scanning electron microscope (SEM) images, in comparison to the uncoated textiles (Figure S1a, Section 2 in the Supporting Information), a greater amount of graphene flakes deposited on the fiber surface during coating (Figure 3d,e) and their restacking through van der Waals forces exerted by the squeeze rollers consequently reduce the resistance of the fabric. We repeated the same process for the other 2D material, i.e., MoS_2_ of the same concentration (10 g L^–1^). Ten textile samples were coated with MoS_2_ from 1 to 10 successive MoS_2_ layers. Both the graphene coated and MoS_2_ coated textiles were utilized as control electrodes for further studies. The SEM images of the MoS_2_-coated textiles also exhibit similar phenomena (Figure S1b,c, Section 2 in the Supporting Information). It is worth noting that, being a semiconductor, MoS_2_-coated textiles do not show any conductivity at the initial coating layers. After 8–9 coating layers, the textiles exhibited a very high resistance of ∼0.8–0.9 GΩ cm^–1^, i.e., very poor conductivity.

After preparing graphene and MoS_2_ coated textiles, we aimed to produce heterostructures based on these two 2D materials. For bilayered structures, we first coated the textiles with MoS_2_ (one to five coating layers), followed by graphene coating (one to five layers). This resulted in 25 configurations (Table S1, Section 1 in the Supporting Information). Figure 3b illustrates the changes in the resistance for the bilayered textiles with varying coating layers in each configuration. The single MoS_2_-single graphene coated textile (M1G1) exhibited a resistance of ∼1401 Ω cm^–1^. As the number of graphene coatings on top of the MoS_2_ layer increased, the sheet resistance continued to decrease. At the second graphene coating (M1G2), the resistance reached ∼483.1 Ω cm^–1^, which further decreased to ∼203.43 Ω cm^–1^ after five graphene coatings (M1G5). This phenomenon can be attributed to the increased deposition of conductive material (Figure 3f) on MoS_2_-coated textiles with each additional graphene layer. We also varied the number of MoS_2_ base coating layers. It was observed that the sheet resistance decreased by ∼80% (from ∼1401 to ∼274.9 Ω cm^–1^) between the M1G1 and M5G1 layered textiles. Ultimately, the configuration with the lowest sheet resistance was achieved with M5G5 layered textiles, measuring ∼110.75 Ω cm^–1^, which was around ∼60% lower than that of the M5G1 layered textiles.

To explore the impact of heterostructures on textiles, we investigated trilayered configurations for achieving conductive e-textiles. Initially, we coated the textiles with graphene using a pad-dry method. Subsequently, MoS_2_ was applied onto the graphene-coated textiles, followed by an additional graphene layer deposition using the same method. This resulted in another 25 configurations (Table S1, Section 1 in the Supporting Information). Figure 3c illustrates the changes in sheet resistance for the trilayered textiles with varying coating layers in each configuration. The single graphene-single MoS_2_-single graphene (G1M1G1) layered textiles exhibited a resistance of ∼789.8 Ω cm^–1^. Increasing the number of MoS_2_ and graphene layers contributed to a reduction in the resistance. As the number of MoS_2_ layers increased between two single graphene layers (G1-M(*x*)-G1 configuration), the resistance progressively decreased. For the G1M5G1 layer, the resistance measured was approximately ∼226.85 Ω cm^–1^, which was ∼71.27% of that of the initial G1M1G1 layer. Increasing the graphene layers also led to a reduction in the resistance. The G5M1G5 layer exhibited a resistance of ∼126 Ω cm^–1^, corresponding to an ∼84% reduction compared to the initial G1M1G1 layer. Furthermore, increasing all coating layers, including the initial graphene, intermediate MoS_2_, and final graphene layers, significantly reduced the resistance. The configuration with the lowest resistance was achieved with G5M5G5, measuring ∼74 Ω cm^–1^, representing an ∼90.6% reduction compared to the initial G1M1G1 layer. Notably, the trilayered structure demonstrated significantly lower resistance compared to the bilayered structures due to an overall greater deposition of active materials (Figure 3g). The flexibility of the several coated e-textiles was also evaluated. The change in their electrical resistances per 12 cm length during bending, compression, and folding were measured. The cord length, which was measured by the grip distance of the sample ends during the experiment, was changed (10 to 120 mm) when the fabrics were bent and compressed. Similar to the graphene coated textiles (Figure S2a left, Section 3, in the Supporting Information), no significant changes in the resistance (Δ*R*/*R*_0_) were observed during bending and compression of the heterostructure coated textiles (Figure 3h). Even during folding–releasing operations, the changes in the resistance of the coated textiles were found to be negligible, proving the outstanding flexibility of heterostructure based textiles (Figure S2a right, Section 3, in the Supporting Information). It is worth noting that no visible changes in appearance or shape or creasing were observed due to those mechanical actions (bending, compression, and folding cycles) of the heterostructure-based wearable e-textiles (Figure S2b–e, Section 3, in the Supporting Information).

### Electrochemical Characterization of Heterostructure-Based Textile Supercapacitors

Initially, we utilized multiple graphene layer coated textiles as electrodes to fabricate a graphene based symmetric textile supercapacitor. A thorough analysis of the cyclic voltammetry (CV), galvanostatic charge–discharge (GCD), and electrical impedance spectroscopy (EIS) was carried out for the capacitor with the highest capacitance (Section 4 in the Supporting Information). The supercapacitor with a single graphene coating (G1 electrodes) exhibited an areal capacitance of ∼7.74 mF cm^–2^ at a scan rate of 1 mV s^–1^. The highest areal capacitance of ∼80.19 mF cm^–2^ at a scan rate of 1 mV s^–1^ was achieved with the textile supercapacitor fabricated using 10 graphene coating layers (G10). Similarly, we also fabricated MoS_2_-based symmetric textile supercapacitors (Section 5 in the Supporting Information). Being a semiconductor, the highest areal capacitance with bare MoS_2_ was achieved with 10 coating layers (M10) at ∼7.1 mF cm^–2^ and a scan rate of 1 mV s^–1^. To enhance the capacitance performance of our textile supercapacitors, we explored a combination of heterostructures by depositing both graphene and MoS_2_ onto the textile electrodes. We initially coated textiles with MoS_2_ at different layer configurations (M*x*, where *x* is the number of coating layers from 1 to 5), followed by graphene coating at different layer configurations (G*x*, where *x* is the number of coating layers from 1 to 5). These bilayered configurations of MoS_2_ and graphene (M*x*-G*x*) coated textiles were used as electrodes for the supercapacitors and exhibited the characteristics of a hybrid supercapacitor (ultracapacitor), combining the principles of double-layer capacitance and pseudocapacitance.

**Figure 4:**
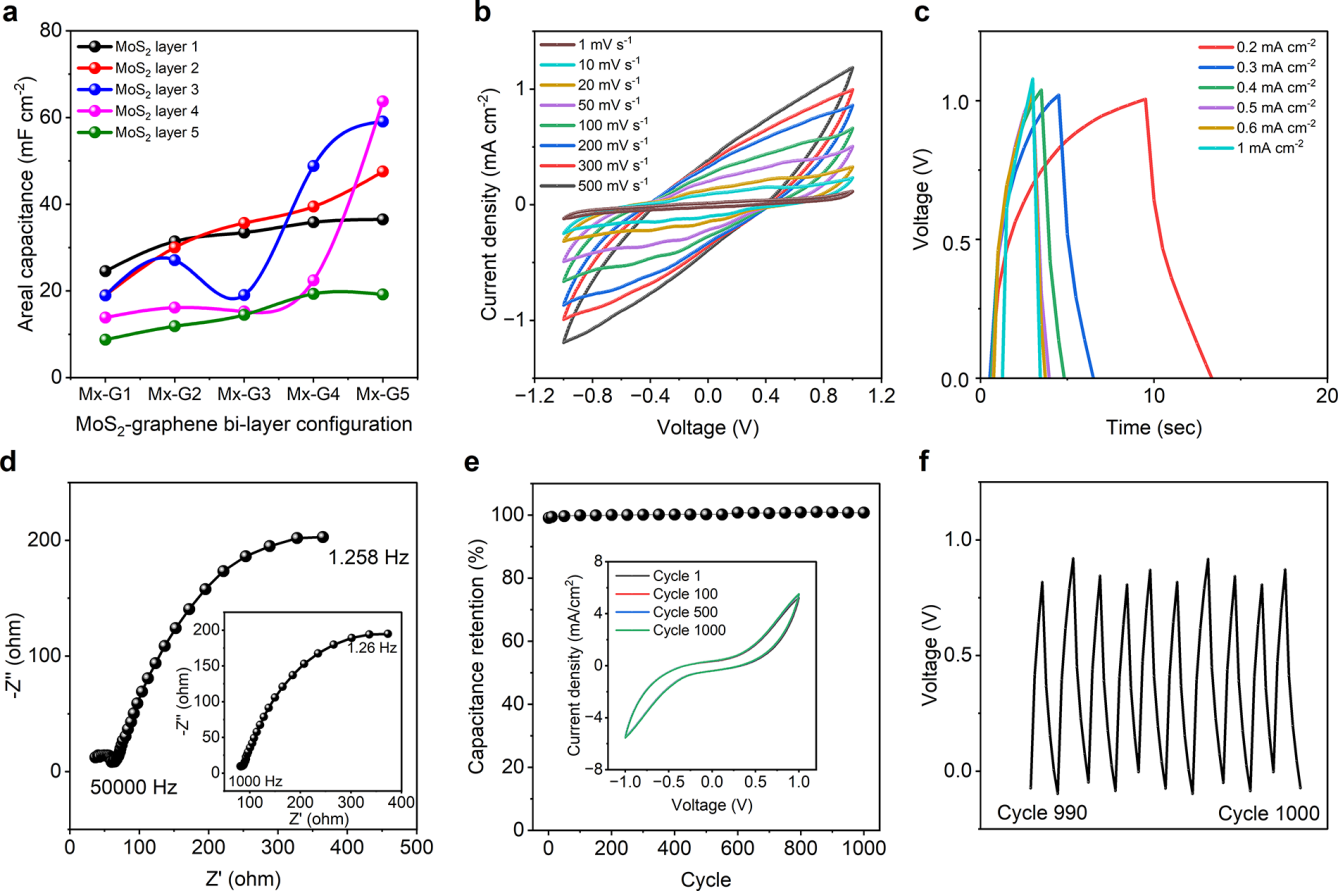
MoS_2_-graphene bilayer coated textile supercapacitor. (a) Change of areal capacitance with increase of coating layers. (b) Cyclic voltammetry curves of the G5M4 coated textile supercapacitor at various scan rates. (c) Charge–discharge profile of the G5M4 coated textile supercapacitor at different current densities. (d) Electrical impedance spectroscopy of the device at high frequency. The inset shows the response of the supercapacitor device at low frequency. (e) Capacitance retention of the G5M4 coated textile supercapacitor device up to 1000 cycles. The inset shows the CV curves at the first, 100th, 500th and 1000th cycles. (f) Cyclic test of the supercapacitor (from 990th to 1000th cycles).

A total of 25 samples were fabricated to investigate the effect of bilayered configurations on the areal capacitance of the textile supercapacitors. Figure 4a demonstrates the change of areal capacitance for different layer configurations. The highest areal capacitance of ∼36.49 mF cm^–2^ was achieved when textiles were coated with a single layer of MoS_2_ followed by five layers of graphene (M1G5 configuration) at a scan rate of 1 mV s^–1^. However, increasing the number of MoS_2_ layers under a single graphene layer led to a decrease in the areal capacitance. The lowest value of ∼8.735 mF cm^–2^ was obtained when textiles were coated with five layers of MoS_2_ under a single graphene layer (M5G1 configuration). Similarly, increasing the number of graphene layers while keeping a single MoS_2_ layer also resulted in increased capacitance up to ∼31.435 mF cm^–2^ for the M1G2 configuration. Among the 25 configurations, the highest areal capacitance of ∼63.73 mF cm^–2^ was achieved when textiles were coated with four layers of MoS_2_ covered by five layers of graphene (M4G5 configuration). Interestingly, further increasing the number of MoS_2_ layers under the graphene layers led to a dramatic reduction in the areal capacitance to ∼19.16 mF cm^–2^. This could be attributed to the excessive amount of graphene interfering with the conductivity of the MoS_2_ flakes. There exists an optimum quantity of both graphene and MoS_2_ for achieving ideal hybrid capacitance behavior. We note that the areal capacitance of the MoS_2_-graphene bilayered electrode based symmetric textile capacitor was almost similar to that of the graphene electrode based symmetric textile supercapacitor, exhibiting 71.62 mF cm^–2^ at 9 coating layers, reaching 80.18 mF cm^–2^ after 10th coating layer (Figure S3a, Section 4, in the Supporting Information). However, a textile supercapacitor composed of only MoS_2_ electrodes exhibited the highest areal capacitance of only 7.1 mF cm^–2^ after 10 coating layers (Figure S4a, section 5, in the Supporting Information).

Several electrochemical tests (CV, GCD, and EIS) were carried out for the best-performing supercapacitor based on M4G5 electrodes. The CV curves exhibited near-rectangular shapes at all tested scan rates, indicating ideal capacitance behavior (Figure 4b). Though for only graphene- and only MoS_2_-based supercapacitors, the current density reaches ∼1.74 mA cm^–2^ (Figure S3b, Section 4, in the Supporting Information) and 1.08 mA cm^–2^ (Figure S4b) respectively, for the bilayered structure, the current density reaches a maximum of only ∼1.19 mA cm^–2^. The charge–discharge profiles (Figure 4c) showed no plateaus or bends, confirming the absence of redox reactions. The slight potential drop observed at the beginning of the discharge curve was due to the device’s ESR, but it did not significantly affect the conductivity or charge barrier of the electrodes. It is to note that, at a current density of 0.2 mA cm^–2^, the charge–discharge took ∼206 s for the graphene-based supercapacitor (Figure S3c, Section 4, in the Supporting Information) and ∼8.5 s for the MoS_2_-based supercapacitor (Figure S4c, Section 5, in the Supporting Information). In contrast, the MoS_2_-graphene bilayered electrode-based textile supercapacitor took ∼13 s at the same current density. The EIS results (Figure 4d) demonstrated an ESR of ∼81.63 Ω at a lower frequency range (1 kHz), which decreased to ∼36.89 Ω at a higher frequency range (50 kHz). The graphene and MoS_2_ exhibited ESRs of ∼37.01 and ∼29.44 Ω at a lower frequency range (1 kHz) and ∼26.78 Ω at a higher frequency range (50 kHz) (Figures S3d and S4d in the Supporting Information). The Nyquist plot exhibited a 45° bend at the low-frequency range, indicating the ideal behavior of a capacitor, Figure 4d.

We evaluated the electrochemical stability of the supercapacitor by examining its long-term charge–discharge behavior at a current density of 1 mA cm^–2^. The device retained its initial capacitance even after 1000 cycles, demonstrating exceptional stability (Figure 4e). The inset in the figure illustrates the CV profile of the supercapacitor up to 1000 cycles, showing no deviations. To further illustrate this stability, Figure 4f displays the charge–discharge curves specifically from the 990th to the 1000th cycle of the GCD tests. The M4G5 supercapacitor, without the use of any current collector, exhibited an impressive areal energy density of ∼35.41 μWh cm^–2^ (∼44.55 μWh cm^–2^ for graphene, ∼3.94 μWh cm^–2^ for MoS_2_) and a power density of 8497.33 μW cm^–2^ (∼581.05 μW cm^–2^ for graphene and ∼3550 μW cm^–2^ for MoS_2_). Additionally, it achieved a specific energy density of 9.32 Wh kg^–1^ (∼12.73 Wh kg^–1^ for graphene and ∼1.59 Wh kg^–1^ for MoS_2_) and a power density of ∼2,236.14 W kg^–1^, (∼166.01 W kg^–1^ for graphene and ∼1420 W kg^–1^ for MoS_2_). The exceptionally high power density shows promise for our textile supercapacitor for next-generation wearable applications.

To achieve higher capacitance performance of our textile supercapacitor, we introduced another initial graphene layer on the previous bilayer configuration, resulting in a trilayered heterostructure component configuration. Initially, we coated textiles with graphene at different layer configurations (G*x*, where *x* is the number of coating layers, 1 to 5). These textiles were then coated with several MoS_2_ layers at varying configurations (M*x*, where *x* is the number of coating layers, 1 to 5). Finally, the graphene-MoS_2_ coated textiles were further coated with graphene at different layer configurations (G*x*, where *x* is the number of coating layers, 1 to 5). Textiles coated with several trilayered configuration of 2D materials (G*x*-M*x*-G*x*) were then utilized as supercapacitor electrodes. Similar to our bilayered structure, the graphene-MoS_2_-graphene trilayered electrodes exhibited a hybrid capacitance behavior when fabricated into a supercapacitor.

**Figure 5:**
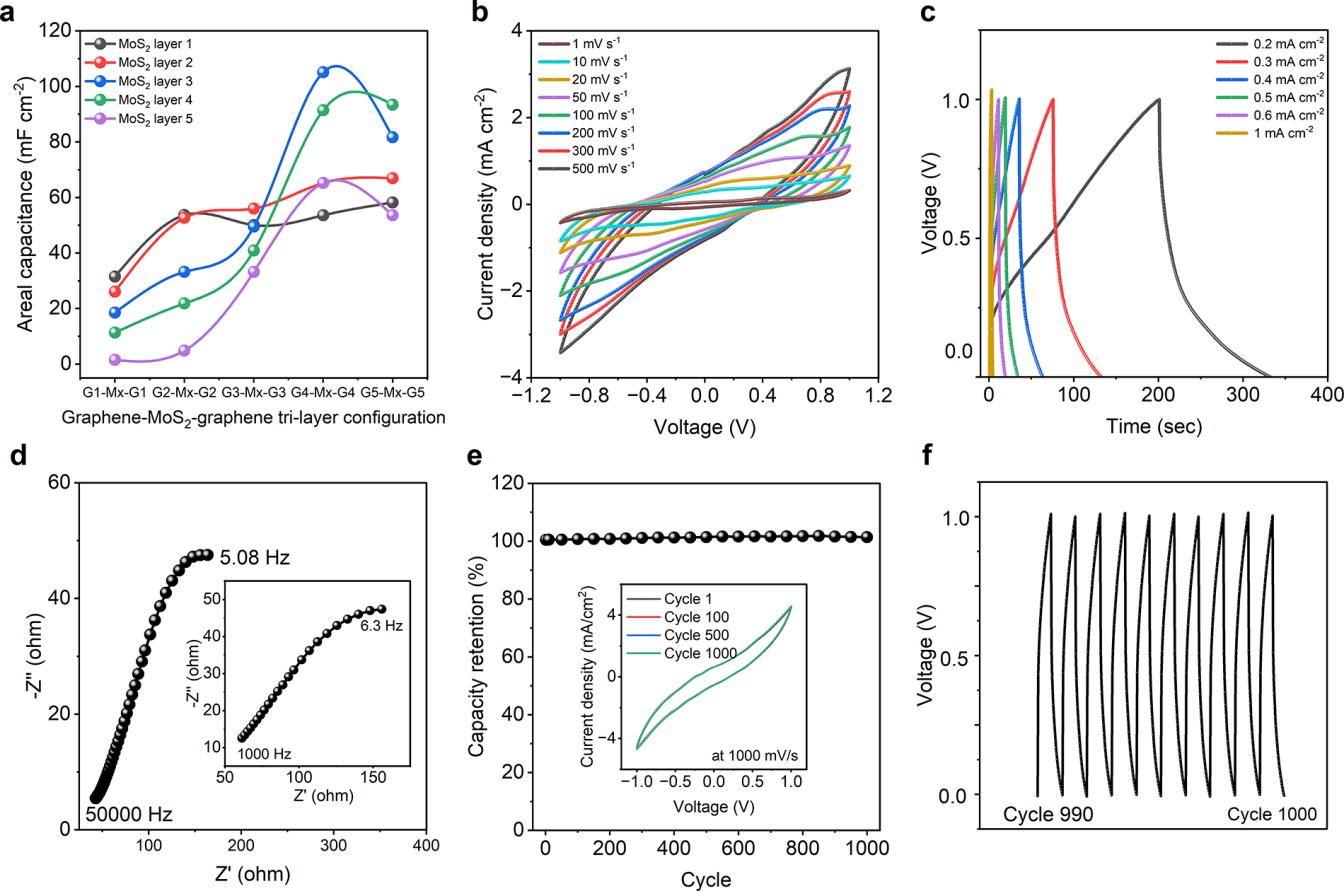
Graphene-MoS_2_-graphene trilayer coated textile supercapacitor (a) Change of areal capacitance with increase of coating layers. (b) Cyclic voltammetry curves of the G4M3G4 coated textile supercapacitor at various scan rates. (c) Charge–discharge profile of the G4M3G4 coated textile supercapacitor at different current densities. (d) Electrical impedance spectroscopy of the device at the high frequency range. The inset shows the response of the supercapacitor device at the low frequency range. (e) Capacitance retention of the G4M3G4 coated textile supercapacitor device up to 1000 cycles. The inset shows the CV curves at first, 100th, 500th and 1000th cycles. (f) Cyclic test of the supercapacitor (from 990th to 1000th cycles).

Figure 5a demonstrates the change in the areal capacitance of the textile supercapacitors fabricated with graphene-MoS_2_-graphene trilayers in various configurations. Like the bilayered structure, we fabricated a total of 25 coated samples to evaluate the effect of trilayered textile electrodes for supercapacitor applications. As observed, supercapacitors fabricated with electrodes containing a single layer of graphene, followed by a single layer of MoS_2_ and a final single layer of graphene (i.e., G1M1G1), exhibited an areal capacitance of ∼31.48 mF cm^–2^ at a scan rate of 1 mV s^–1^. Similar to the bilayered textile SCs, increasing the number of graphene layers enhanced the areal capacitance, reaching values of ∼53.58 and ∼58.16 mF cm^–2^ at the same scan rate for G4M1G4 and G5M1G5 configurations, respectively. Interestingly, increasing the number of MoS_2_ layers between the top and bottom graphene layers further improved the supercapacitor’s areal capacitance. With one MoS_2_ layer in between top 4-layer and bottom 4-layer graphene the capacitance was ∼53.58 mF cm^–2^, which increased to ∼65.21 mF cm^–2^ with an increase of another layer of MoS_2_. The G4M3G4 configuration achieved the highest value of ∼105.08 mF cm^–2^ at a scan rate of 1 mV s^–1^. However, further increases in the number of MoS_2_ layers reduced the capacitance to ∼91.37 mF cm^–2^ (G4M4G4) and ∼65.21 mF cm^–2^ (G4M5G4) at the same scan rate. This phenomenon could be attributed to the fact that the addition of a layer of MoS_2_ contributes to fill the voids on the graphene surface, providing additional active sites for charge storage (pseudocapacitance in the case of MoS_2_) and improving the overall capacitance. After a certain limit, the excess amount of MoS_2_ starts to interfere with the overall charge storage of the structure. Since MoS_2_ is a semiconductor material, the presence of excess amount might have obstructed the capacitive behavior of the EDL graphene material as well as the overall heterostructure.

A similar trend was observed for the G5 configuration of trilayered structures. The supercapacitor with a G5M1G5 electrode exhibited an areal capacitance of ∼58.16 mF cm^–2^ at a scan rate of 1 mV s^–1^, which increased to ∼93.4 mF cm^–2^ at the same scan rate. However, further increases in the number of MoS_2_ layers reduced the capacitance to ∼53.58 mF cm^–2^. This phenomenon can be attributed to the amount of active material loaded into each coating layer. For example, the total number of coating layers was 3 for G1M1G1 (∼31.48 mF cm^–2^), 11 for G5M1G5 (∼58.16 mF cm^–2^), 11 for G4M3G4 (∼105.08 mF cm^–2^), and 14 for G5M4G5 (∼93.4 mF cm^–2^). Textiles can absorb active material up to a certain limit, and beyond saturation, additional coating hinders performance rather than adding functionality. When considering the 11-layered structures (G5M1G5 and G4M3G4), it is evident that despite the reduced amount of highly conductive graphene, the introduction of a semiconductor material within a certain limit increases the capacitance of the fabricated supercapacitor by ∼80.69%. The effect of MoS_2_ presence in the supercapacitor is also evident in electrodes fabricated with G4M1G4 and G4M3G4 structures. The addition of 2 MoS_2_ coating layers between the G4-M*x*-G4 configuration contributes to an enhancement of ∼96.14% in areal capacitance.

We conducted a comprehensive analysis of the electrochemical performance (CV, GCD, and EIS) of our highest-performing supercapacitor configuration (G4M3G4). The CV curves exhibited nearly rectangular shapes at all scan rates (Figure 5b), indicating ideal capacitance behavior. As previously discussed, in comparison to the other configurations, the current density reaches a maximum of ∼3.13 mA cm^–2^. The absence of redox peaks within the tested electrochemical windows suggests complete coverage of cotton fibers by the graphene-based ink. The charge–discharge profile (Figure 5c) of the G4M3G4 supercapacitor showed no visible plateaus or bends associated with the redox reactions. The minor potential drop observed initially can be attributed to the energy consumption of the device’s equivalent series resistance (ESR), which still ensures good conductivity and low charge barrier of the electrodes. It should be noted that the charge–discharge time was extended up to 332 s compared to other heterostructure supercapacitors at the same current density of 0.2 mA cm^–2^. The EIS analysis revealed a low equivalent series resistance (ESR) of ∼61.47 Ω at a lower frequency range (1 kHz) that decreased to ∼42.87 Ω at a higher frequency range (50 kHz), as indicated by the Nyquist plot (Figure 5d). The proximity of the vertical lines to the imaginary axis suggests that the supercapacitor behavior closely resembles that of an ideal capacitor. We also investigated the electrochemical stability of the supercapacitor through long-term charge–discharge curves at a current density of 1 mA cm^–2^. The device maintained its initial capacitance even after 1000 cycles, demonstrating excellent stability (Figure 5e). The inset in Figure 5e depicts the CV profile of the supercapacitor up to 1000 cycles, showing no deviation. Furthermore, Figure 5f presents the charge–discharge curves of the 990th to 1000th cycle in the GCD tests. The G4M3G4 supercapacitor achieved the highest areal energy density of ∼58.37 μWh cm^–2^ (∼35.41 μWh cm^–2^ for M4G5 bilayered supercapacitor, ∼44.55 μWh cm^–2^ for graphene, and ∼3.94 μWh cm^–2^ for MoS_2_ supercapacitor). However, the power density was 1604.27 mW cm^–2^ (though the highest was for the M4G5 bilayered supercapacitor at ∼8497.33 μW cm^–2^, with ∼581.05 μW cm^–2^ for graphene and ∼3550 μW cm^–2^ for MoS_2_ supercapacitor). The specific energy density of ∼14.59 Wh kg^–1^ was achieved, whereas it was ∼9.317 Wh kg^–1^ for M4G5 bilayered, ∼12.73 Wh kg^–1^ for graphene, and 1.59 Wh kg^–1^ for MoS_2_ supercapacitor. The power density was reported as ∼401.06 W kg^–1^ (∼2,236.14 W kg^–1^ for M4G5 bilayer ∼166.01 W kg^–1^ for graphene, and ∼1420 W kg^–1^ for MoS_2_ supercapacitor). The exceptionally high energy and power densities show promise for our textile supercapacitor for next-generation wearable applications.

**Figure 6:**
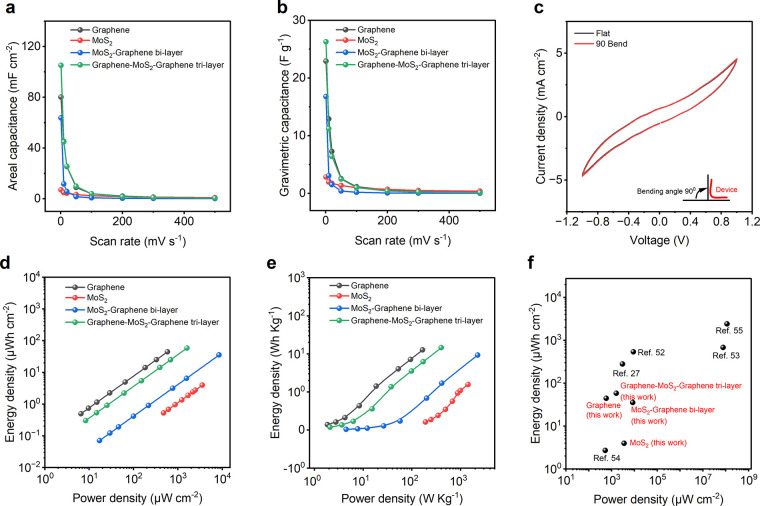
Performance of the 2DM heterostructure based textile supercapacitors. (a) Areal capacitance of 4 as-fabricated textile supercapacitors at different scan rates. (b) Gravimetric capacitance of 4 as-fabricated textile supercapacitors at different scan rates. (c) CV curves of the G4M3G4 supercapacitor in flat and bent (at 90° angle) condition . (d) Ragone plot showing comparison of 4 as-fabricated supercapacitor performanced (in terms of areal energy and power density). (e) Ragone plot showing comparison of 4 as-fabricated supercapacitor performances (in terms of specific energy and power density). (f) Ragone plot showing comparison of 4 as-fabricated supercapacitor performances with others in literature.

Figure 6 presents a performance analysis of our fabricated supercapacitors. Areal and gravimetric capacitances, as a function of scan rate, are depicted in Figure 6a,b, respectively. It can be observed that higher scan rates result in lower capacitance, possibly due to insufficient time for electrolytes to adsorb and desorb on the electrode surface.^50^ At lower scan rates, electrolyte ion diffusion becomes more efficient, reaching both the external surface and inner active sites of the electrode material.^51^ In Figure 6c, the CV curves of the device are shown when it is both flat and bent at a 90° angle, demonstrating the device’s stable electrochemical performance, even under bending conditions. The Ragone plots in Figure 6d,e showcase the energy and power densities of our fabricated supercapacitors. Notably, our graphene-MoS_2_-graphene trilayered textiles exhibit impressive energy and power densities of ∼58.38 μWh cm^–2^ and ∼1604.27 μW cm^–2^, respectively. These values are either superior or comparable to those reported in previous studies (Figure 6f) on graphene-based wearable supercapacitors.^27,52−60^

### Small-Scale Integration of Textile Supercapacitors

**Figure 7:**
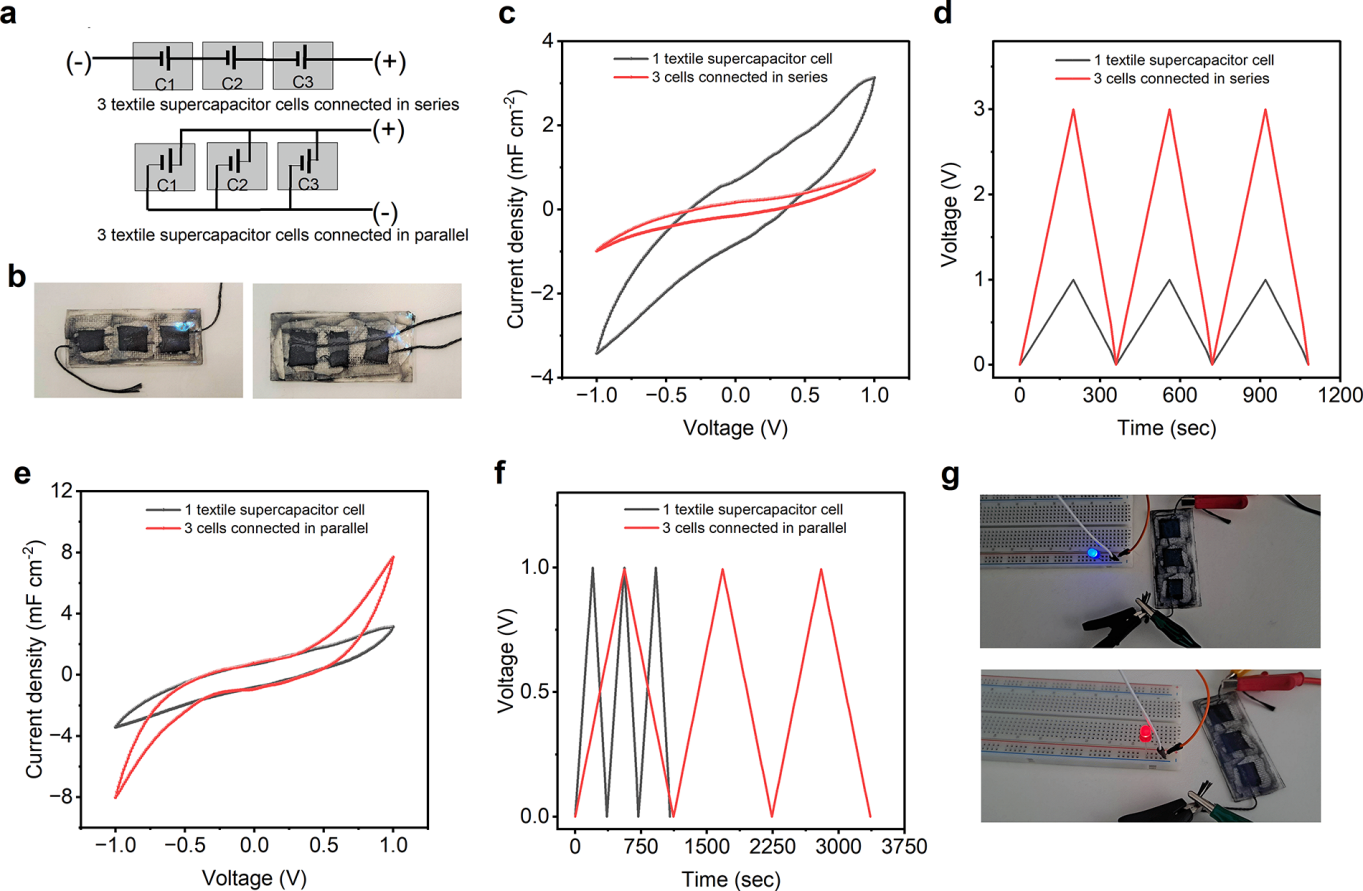
Small-scale integration of 2DM heterostructure based textile supercapacitor. (a) Schematic of 3 supercapacitor cells in series (top) and parallel (bottom) connection. (b) Three as-fabricated textile supercapacitor cells connected in series (left) and parallel (right) connection. (c) CV profile of 1 supercapacitor cell versus 3 supercapacitor cells connected in series (at scan rate 500 mV s^–1^). (d) GCD curve of 1 supercapacitor cell versus 3 supercapacitor cells connected in series (at current density 0.2 mA cm^–2^). (e) CV profile of 1 supercapacitor cell versus 3 supercapacitor cells connected in parallel (at scan rate 500 mV s^–1^). (f) GCD curve of 1 supercapacitor cell versus 3 supercapacitor cells connected in parallel (at current density 0.2 mA cm^–2^). (g) Three supercapacitor cells connected in series powering LEDs.

In practical applications, a single supercapacitor often does not provide sufficient voltage and current to power circuits effectively.^61^ Therefore, multiple supercapacitors are commonly connected in series and/or in parallel (Figure 7a) to form a “bank” with a specific voltage and capacitance.^8^Figure 7b shows the small-scale connection of 3 supercapacitors in series (left) and parallel (right). The conductive current collector threads were employed as connection wires for the supercapacitors. Figure 7c shows the CV profiles, and Figure 7d shows the GCD curves of the three series-connected textile supercapacitors. It is evident that the series connection increases the voltage window from 1 to 3 V, with a reduction in capacitance to approximately one-third of a single device. On the other hand, Figure 7e displays the CV profiles, and Figure 7f presents the GCD curves for the three parallel-connected textile supercapacitors. In contrast to the series connection, the parallel connection exhibits nearly 3 times higher capacitance compared to a single device while maintaining the same operating voltage window. Furthermore, the parallelly connected supercapacitors were capable of powering different colored LEDs with varying voltage requirements, as depicted in Figure 7g. This highlights the capability of our textile-based flexible supercapacitors to meet the increasing energy needs of wearable electronics in the future.^62−64^

## Conclusions

In this work, we report the scalable production of two very promising 2D materials, namely graphene and MoS_2_, through a microfluidization technique. Considering the design flexibility of 2D materials for tuning their electronic properties, we report a graphene and MoS_2_ heterostructure for wearable e-textiles based supercapacitor fabrication. Supercapacitors were fabricated from graphene-coated textiles, MoS_2_-coated textiles, MoS_2_-graphene bilayer coated textiles, and graphene-MoS_2_-graphene trilayered textiles. The highest areal capacitance was obtained from the supercapacitor fabricated from the graphene-MoS_2_-graphene trilayered textile electrodes of ∼105.08 mF cm^–2^ at a scan rate of 1 mV s^–1^. The energy and power densities were ∼58.38 μWh cm^–2^ and ∼1604.27 μW cm^–2^, respectively. Though the energy density was highest for the trilayered electrode-based supercapacitor, the highest power density was achieved for the MoS_2_-graphene bilayered electrode based supercapacitor. The supercapacitors showed an outstanding capacitance retention (∼100%) after 1000 cycles. The integration of 3 as-fabricated supercapacitors enable powering up an LED. Overall, these outstanding performances exhibit prospects of 2D material heterostructures to revolutionize the e-textiles , especially the field of energy storage. Industrially the most-known and highly scalable coating method was exploited , which demonstrates the potential of reported fabrication process for the large-scale production of 2D heterostructure based wearable e-textile supercapacitors.

## Experimental Methods

### Materials

A water-based graphene and MoS_2_ dispersion (100 g L^–1^) was prepared using a microfluidization technique. Natural flake graphite (average lateral size ∼50 μm) and MoS_2_ were purchased from Sigma-Aldrich, UK. Sodium deoxycholate (SDC) powder was purchased from Sigma-Aldrich, UK, and used as received. 100% cotton fabric (desized, scoured, and bleached, which creates a ready-to-dye fabric) was manufactured at Square Fashions Limited (Bangladesh).

### Microfluidic Exfoliation of Graphene and MoS_2_

Microfluidization is characterized by a homogenization technique; a high pressure (up to 207 MPa)^65^ is applied to a fluid, which forces the liquid to pass through a microchannel (diameter, *d* < 100 μm). This technique offers the advantage of applying high γ̇ > 10^6^ s^–1^ to the whole dispersion,^66^ unlike just locally as in the case of sonication and shear-mixing. Previous studies reported a microfluidization technique for a range of purposes such as the production of polymer nanosuspensions,^65^ liposome nanoparticles,^67^ aspirin nanoemulsions,^68^ oil-in-water nanoemulsions,^40^ and deagglomeration and dispersion of carbon nanotubes.^69^ In this study, we used a microfluidization technique to exfoliate graphene and MoS_2_ in a scalable quantity following previously reported methods.^38,44^ Briefly, 50 g of graphite powder and 10 g of SDC were placed in a glass bottle and mixed with 500 mL of deionized (DI) water. The mixture was then sonicated for 30 min using an ultrasound bath to allow homogeneous dispersion and placed in an input reservoir of a Microfluidizer (M-110P Microfluidizer, Microfluidics Corp, USA). The dispersion was slowly passed through “Z-type” microfluidic channels of ∼200 and ∼87 μm diameter with diamond construction at high pressure (∼200 MPa). This allows the exfoliation of graphite to few-layer graphene at 100 mL min^–1^ flow under a high shear rate (∼10^8^ s^–1^) with 100% exfoliation yield. The exfoliated dispersion was then passed through a cooling channel surrounded by cold water (∼25 °C) to prevent overheating of the dispersion and collected. This process was repeated 20 times to produce graphene flakes. MoS_2_ was also produced following the same method. The obtained dispersion was used as a conductive ink for textile coating.

### Scalable Fabrication of Conductive Textiles from 2D Materials Heterostructure

Textile fabrics were padded one dip and one nip through graphene dispersions to a wet pick-up of ∼80% on the weight of the fabric (o.w.f.). The wet pick-up % was calculated using the following formula:
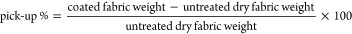
A simple laboratory-scale padder BVHP 2 Bowl (Roaches, UK) was used for coating. The padding roller was set at a speed of 1 m min^–1^ with a pressure of 0.74 bar. The padded fabrics were subsequently dried at ∼100 °C for 5 min in a Mini Thermo Oven, Type 350 Special (Roaches, UK), and studied for e-textile application. Several coated samples were prepared using multiple (1–10) padding passes to establish any improvement in the electrical conductivity of the coated fabrics. We carried out a similar process for MoS_2_ as a monomaterial control sample. For the MoS_2_-graphene bilayered textiles, we coated the fabrics with MoS_2_ first at different configurations (from 1 to 5 layers). The coated fabrics were then again coated with graphene dispersions (from 1 to 5 layers). A total of 25 samples was thus prepared. The resistance of each sample was assessed to check the improvement in the electrical conductivity with the number of coating layers.

For the trilayered structure of graphene and MoS_2_, the fabrics were first coated with graphene dispersion (up to 5 layers). The samples were then coated with MoS_2_ with the same layer variation (from 1 to 5 layers). The coated samples were further coated with graphene, keeping the layer variation similar (from 1 to 5 layers). Similar to bilayered structures, a total of 25 samples were produced for obtaining the optimum conductivity of the coated textiles. The total configuration of samples is given in Table S1. The surface topographies of the control cotton fabric, graphene coated fabric, MoS_2_ coated, MoS_2_-graphene bilayer coated, and graphene-MoS_2_-graphene trilayer coated fabric were analyzed using a FEI Quanta 650 field emission scanning electron microscope (SEM).

### Supercapacitor Device Fabrication

The 2D material heterostructure coated textiles were used as electrodes, and the conductive textile thread was used as the current collector. The coated electrodes were coated with a hydrogel-polymer electrolyte, poly(vinyl alcohol) (PVA) doped with H_2_SO_4_. The H_2_SO_4_-PVA gel electrolyte was prepared as follows: 1 g of H_2_SO_4_ was added to 10 mL of deionized water, and then 1 g of PVA (molecular weight: 89000–98000) was added. The whole mixture was then heated to 85 °C with stirring until the solution became clear. The electrolyte was drop-cast and left to dry overnight under ambient conditions to ensure that the electrolyte completely wetted the electrode and to allow for evaporation of any excess water. The textile electrodes were then sandwiched with a textile separator to form a textile supercapacitor.

### Characterization of Supercapacitors

The electrochemical performances of the printed devices were investigated by cyclic voltammetry (CV), galvanostatic charge/discharge (GCD) tests, and electrochemical impedance spectroscopy (EIS). The electrochemical measurements were performed on an Iviumstat Electrochemical Interface. The CV tests were carried out in the potential range from −1.0 to 1.0 V at different scan rates. Galvanostatic charge–discharge measurements were at different current densities in the potential range of 0–1 V.

The ability to collect and store energy in the form of electrical charge per unit mass, namely, gravimetric capacitance (F g^–1^), was calculated as per the following formulas:
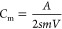

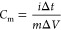


The charge storage ability per unit area, namely areal capacitance (F cm^–2^), was calculated as per the following formulas:
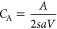

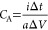
The amount of energy able to be delivered, i.e., energy density (Wh kg^–1^), and how fast the energy can be delivered, namely, power density (W kg^–1^), were calculated as per the formulas
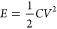

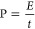
where *I* = current density, *V* = voltage window, *i* = discharging current, Δ*v* = discharge voltage, Δ*t* = discharge time, *A* = integrated area of the CV curve, *s* = scan rate (mV s^–1^), and *m* = mass of the electroactive material on both electrodes.

## Supporting Information Available

Configurations of different coated samples (graphene, MoS_2_, MoS_2_-graphene bilayer, and graphene-MoS_2_-graphene trilayer), SEM images of uncoated, MoS_2_ 1 pass coated, and MoS_2_ 10 pass coated, flexibility of the graphene-coated textiles during bending and compression, flexibility of graphene coated, MoS_2_-graphene bilayer coated, and graphene-MoS_2_-graphene trilayer coated textiles during folding–releasing cycles, graphene-coated textile and fabricated supercapacitor, MoS_2_-coated textile and fabricated supercapacitor, MoS_2_-graphene bilayer coated textile and fabricated supercapacitor, graphene-MoS_2_-graphene trilayer coated textile and fabricated supercapacitor, characterization of graphene-coated textile supercapacitor, characterization of MoS_2_-coated textile supercapacitor, and comparison of the capacitance performance of the heterostructure textile based supercapacitor with others in the literature (PDF)
