# Supplementary material for: Scalable Production of 2D Material Heterostructure Textiles for High-Performance Wearable Supercapacitors
Source: ACS Nano. 2023 Sep 11;17(18):18481–93. doi: 10.1021/acsnano.3c06181 (PMC10540263; doi:10.1021/acsnano.3c06181)
Supplement: Supplementary file 1 — nn3c06181_si_001.pdf [file nn3c06181_si_001.pdf]

Supporting Information

Scalable Production of 2D Material Heterostructure Textiles for High Performance Wearable Supercapacitors

Md Rashedul Islam[1], *Shaila Afroj*[1,2]* *and Nazmul Karim*[1,2,3]*

[1]Centre for Print Research (CFPR), University of the West of England (UWE) Bristol, Frenchay Campus, Bristol, BS16 1QY, UK

[2]National Graphene Institute (NGI), University of Manchester, Oxford Road, Manchester M13 9PL, UK

[3]Advanced Textiles Research Group (ATRG), Nottingham Trent University, Shakespeare Street, Nottingham, NG1 4GG, UK

E-mail: shaila.afroj@uwe.ac.uk and nazmul.karim@ntu.ac.uk

Supporting information 1.

Configuration of coating samples

Table S1. Graphene (G)- MoS_2 (M) – Graphene (G) layer deposition

Configurations	G-M-G	G-M-G	G-M-G	G-M-G	G-M-G
Graphene coating layers	1-0-0	2-0-0	3-0-0	4-0-0	5-0-0
	6-0-0	7-0-0	8-0-0	9-0-0	10-0-0
MoS_2 coating layers	0-1-0	0-2-0	0-3-0	0-4-0	0-5-0
	0-6-0	0-7-0	0-8-0	0-9-0	0-10-0
MoS_2-graphene bi-layers	0-1-1	0-2-1	0-3-1	0-4-1	0-5-1
	0-1-2	0-2-2	0-3-2	0-4-2	0-5-2
	0-1-3	0-2-3	0-3-3	0-4-3	0-5-3
	0-1-4	0-2-4	0-3-4	0-4-4	0-5-4
	0-1-5	0-2-5	0-3-5	0-4-5	0-5-5
Graphene-MoS_2-graphene tri-layers	1-1-1	1-2-1	1-3-1	1-4-1	1-5-1
	2-1-2	2-2-2	2-3-2	2-4-2	2-5-2
	3-1-3	3-2-3	3-3-3	3-4-3	3-5-3
	4-1-4	4-2-4	4-3-4	4-4-4	4-5-4
	5-1-5	5-2-5	5-3-5	5-4-5	5-5-5

Supporting information 2.

Scanning Electron Microscope images of the coated textiles

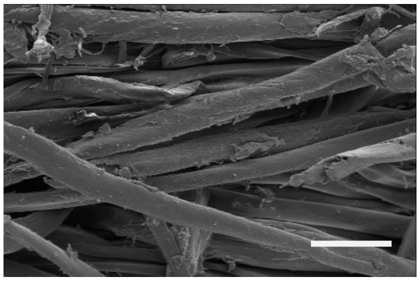

a. Un-coated cotton textiles (x 1000)

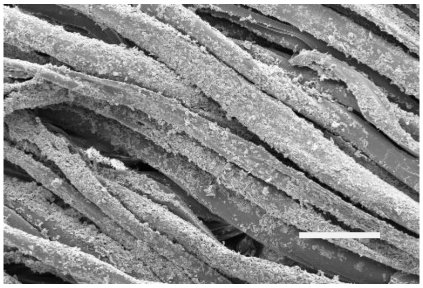

b. MoS_2 1 coated textiles, M1 (x 1000)

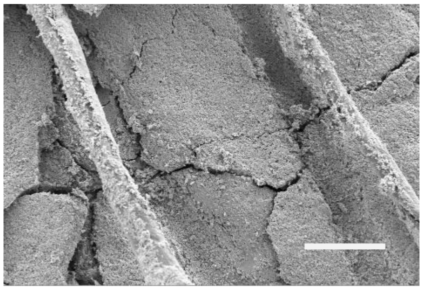

c. MoS_2 10 coated textiles, M10 (x 1000)

Figure S1. *Scanning electron microscope (SEM) image of the a. Un-coated cotton textiles, b. MoS_2 1layer coated textiles c. MoS_2 10-layer coated textiles (Scale bar: 40 μm)*

Supporting information 3.

Flexibility of coated textiles

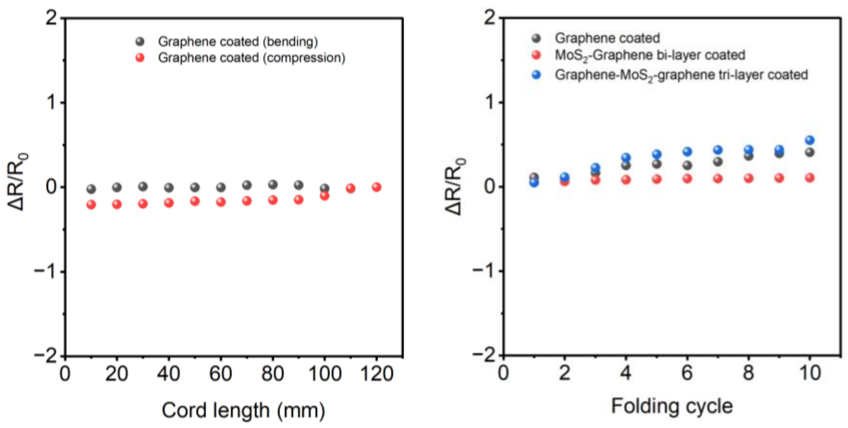

a. The variation in resistance of graphene coated textiles during bending and compression (left) and the variation in resistance of graphene coated, MoS_2-graphene coated and graphene-MoS_2-graphene coated textiles during 10 folding−releasing cycles (right)

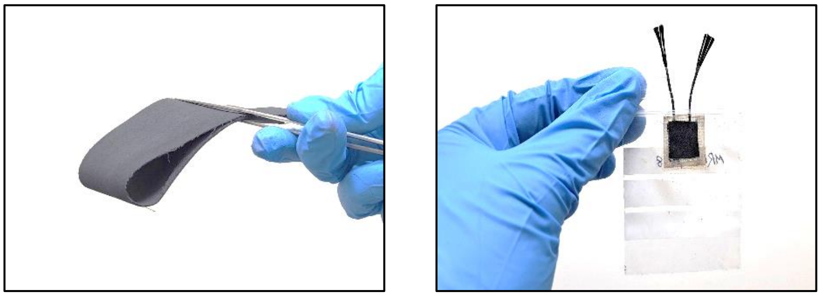

b. Graphene coated textiles and fabricated supercapacitor (1 cm × 1cm)

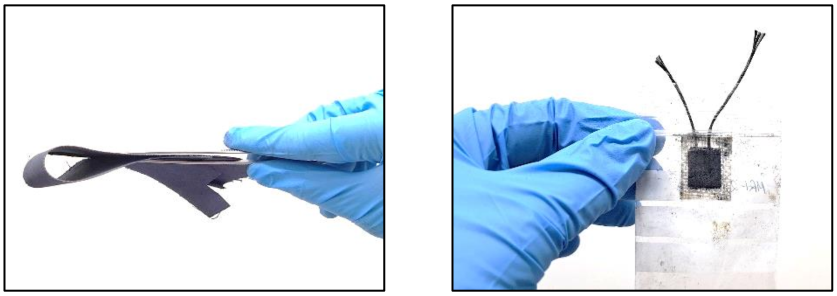

c. MoS_2 coated textiles and fabricated supercapacitor (1 cm × 1cm)

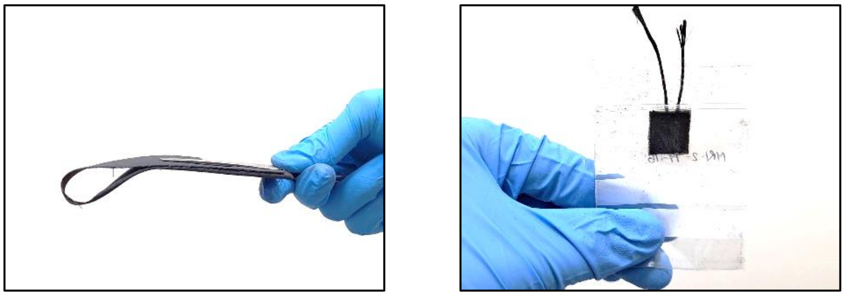

d. MoS_2-graphene bi-layer coated textiles and fabricated supercapacitor (1 cm × 1cm)

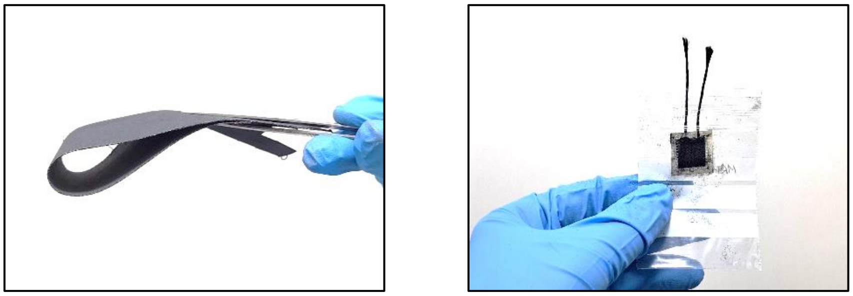

e. Graphene-MoS_2-graphene tri-layer coated textiles and fabricated supercapacitor (1 cm × 1cm)

Figure S2. *Flexibility of the 2D materials-heterostructure coated textiles and as-fabricated supercapacitors*

Supporting information 4.

Electrochemical characterization of graphene-based textile supercapacitors

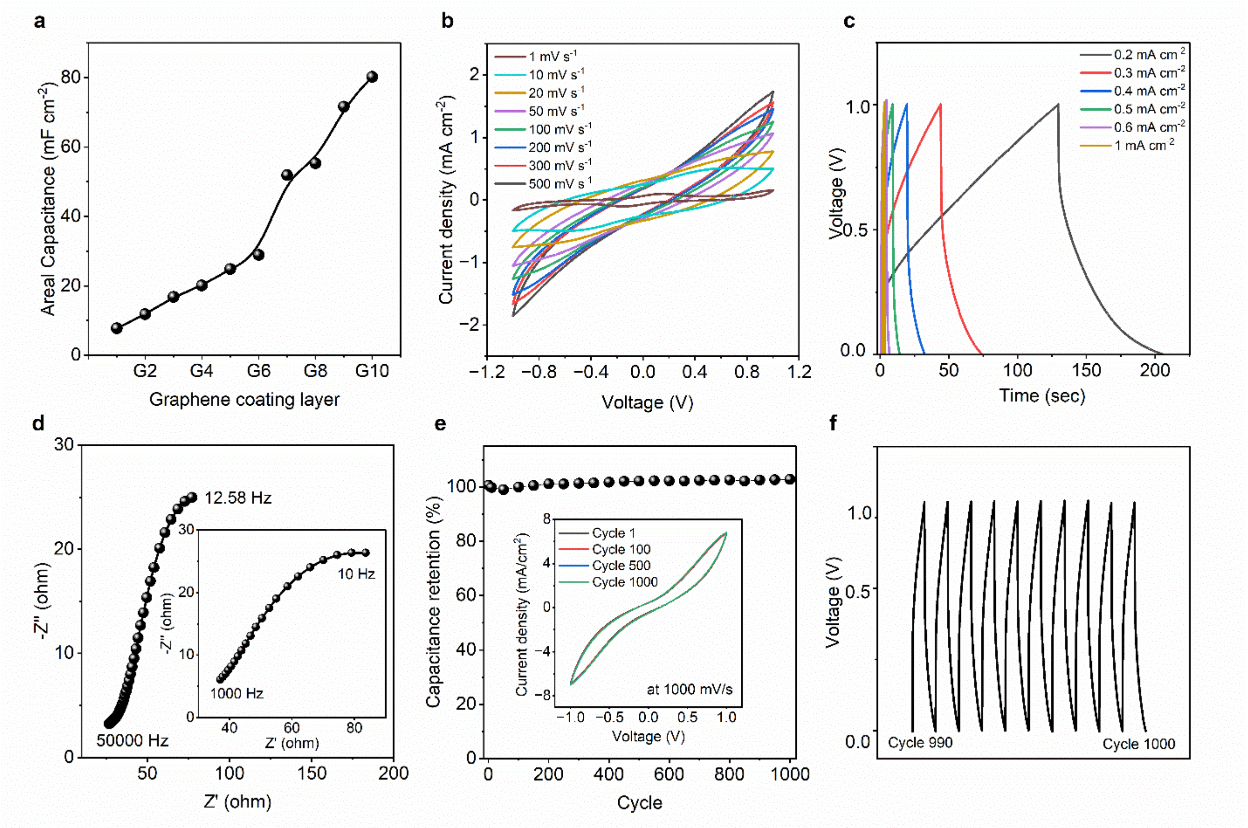

Figure S3. *Graphene-coated textile supercapacitor a. Change of areal capacitance with increase of graphene coating layers b. Cyclic voltammetry curves of the G10 coated textile supercapacitor at various scan rates c. Charge-discharge profile of the G10 coated textile supercapacitor at different current densities d. Electrical impedance spectroscopy of the device at high frequency range (inset shows the response of the supercapacitor device at low frequency range) e. Capacitance retention of the G10 coated textile supercapacitor device up to 1000 cycles, inset shows the CV curves at 1st, 100th, 500th and 1000th cycles and f. cyclic test of the supercapacitor (from 990th to 1000th cycles).*

For the fabrication of our graphene coated symmetric textile supercapacitor, we utilized textiles coated with multiple graphene layers electrodes. The supercapacitor exhibited the double layer capacitance behavior. **Figure S3a** shows the areal capacitance of the textile supercapacitors fabricated with varying graphene coating layers. The supercapacitor with a single graphene coating (G1 electrodes) exhibited an areal capacitance of ~7.74 mF cm^{-2} at a scan rate of 1 mVs^{-1}. As the number of graphene layers increased, the areal capacitance also increased. This can be attributed to the larger amount of active material available for exhibiting electrical double layer capacitance, resulting in an increased capacitance of the device. The highest areal capacitance of ~80.19 mF

cm^{-2} at a scan rate of 1 mVs^{-1} was achieved with the textile supercapacitor fabricated using 10 graphene coating layered (G10).

The electrochemical performance of the best performing (G10) sandwiched shaped supercapacitor was analyzed using cyclic voltammetry (CV), galvanostatic charge-discharge (GCD) and electrical impedance spectroscopy (EIS) tests. CV curves (Figure S3b) exhibited near rectangular shapes at all tested scan rates, indicating ideal double-layer capacitance. The absence of any redox peaks suggested complete coverage of cotton fibers by the graphene-based flakes within the tested electrochemical windows. The charge-discharge profile of the G10 supercapacitor (Figure S3c) displayed triangular shapes, indicative of ideal double-layer behavior with balanced charge and discharge times. No plateaus or bends were observed, indicating the absence of redox reactions. A slight potential drop at the beginning of the discharge curve, known as the IR drop (I = current and R = inner resistance), was attributed to the energy consumption of a device's equivalent series resistance (ESR). The ESR of the G10 supercapacitor device was ~37.01 Ω at a lower frequency (1 kHz) and decreased to ~ 26.78 Ω at a higher frequency range (50 kHz), as indicated by the Nyquist plot of the EIS (Figure S3d) at an overall device area is of 1 cm^2. The ESR, of the device is the value of the real impedance Z' where Z'' close to zero at high frequency region. The inset shows the vectors plotted by the Z' and -Z" of device shows 45 degrees bend at low frequency range which is the ideal behavior of a double layer capacitor. It is at this point where diffusion dominates the behavior of the supercapacitor.

The electrochemical stability of the supercapacitor was also investigated based on long-term charge-discharge curves at a current density of 1 mA cm^{-2}. It showed that the G10 supercapacitor maintained its initial capacitance even after 1,000 cycles, demonstrating excellent stability (Figure S3e). The CV profile of the supercapacitor remained consistent throughout the cycles (inset). Figure S3f shows the charge-discharge curves of the 990th to 1000th cycle in the GCD tests. The G10 supercapacitor, without the use of a current collector, exhibited a high areal energy density of ~44.55 µWh cm^{-2} and a high-power density of ~581.05 µW cm^{-2}. The specific energy density and power densities were calculated as ~12.73 Wh kg^{-1} and ~166.01 W kg^{-1}, respectively.

Supporting information 5.

Electrochemical characterization of MoS_2-textile supercapacitors

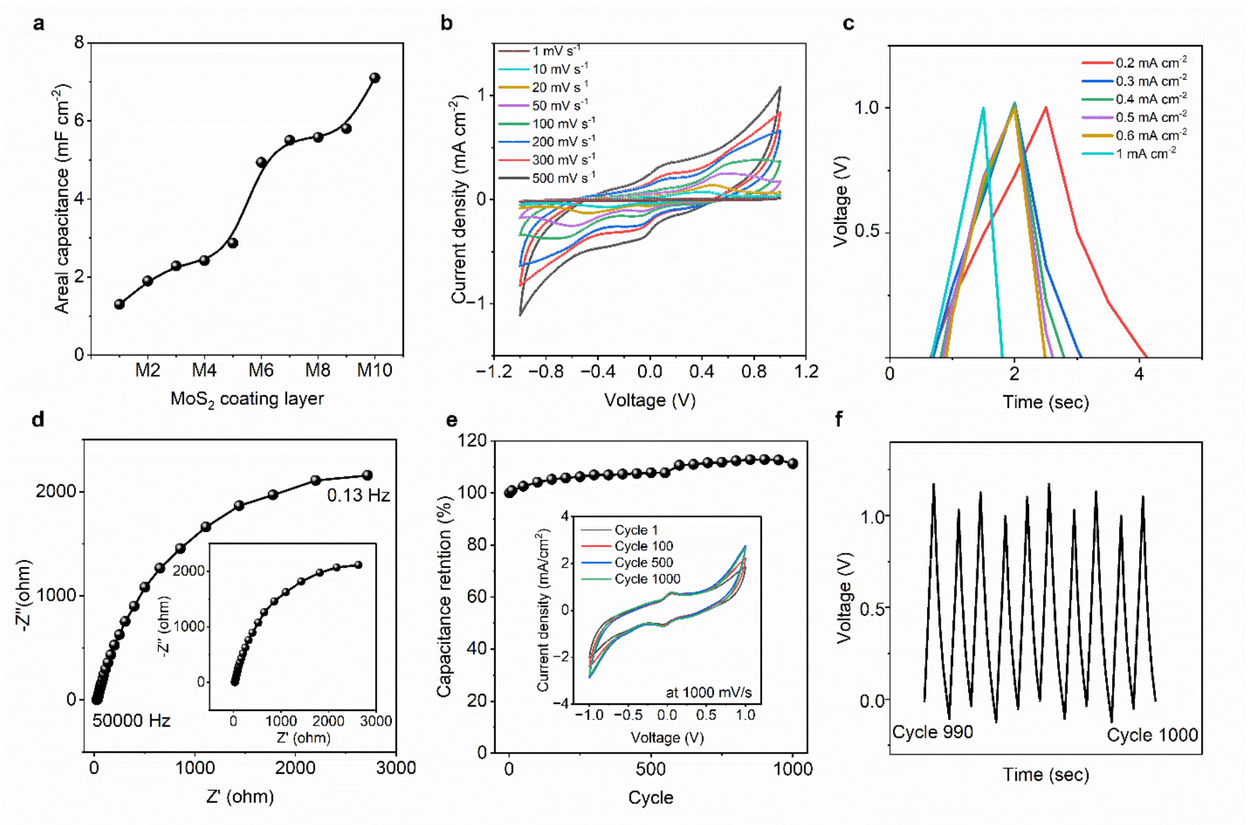

Figure S4. *MoS_2-coated textile supercapacitor a. Change of areal capacitance with increase of MoS_2 coating layers b. Cyclic voltammetry curves of the M10 coated textile supercapacitor at various scan rates c. Charge-discharge profile of the M10 coated textile supercapacitor at different current densities d. Electrical impedance spectroscopy of the device at high frequency range (inset shows the response of the supercapacitor device at low frequency range) e. Capacitance retention of the M10 coated textile supercapacitor device up to 1000 cycles, inset shows the CV curves at 1st, 100th, 500th and 1000th cycles and f. cyclic test of the supercapacitor (from 990th to 1000th cycles).*

We also fabricated symmetric textile supercapacitors coated with successive MoS_2 layers. MoS_2-based textile electrodes store charge through faradic redox reaction, known as pseudo capacitance. The areal capacitance of the textile supercapacitors fabricated with different MoS_2 coating layers (**Figure S4a)** increased with the number of MoS_2 layers. MoS_2 possess graphene-like structure which exhibits pseudocapacitive phenomenon for charge storage. More MoS_2 layers increased the functional material for charge storage, resulting in improved areal capacitance. The highest areal capacitance with bare MoS_2 was achieved with 10 coating layers (M10) at~ 7.1 mF cm^{-2} and a scan rate of 1 mVs^{-1}.

The further analysis of the electrochemical performance (CV, GCD and EIS) the best performing sandwich-shaped supercapacitor (M10) shows quasi-rectangular CV curves at all tested scan rates, exhibiting ideal pseudo-capacitance, Figure S4b. The charge-discharge profiles (Figure S4c) exhibited ideal capacitive behavior with triangular shapes and equal charge and discharge times. No visible plateaus or bends were observed, indicating the absence of redox reactions. The absence of IR drops at the beginning of the discharge curve indicated good conductivity and low charge barriers at the electrodes. The ESR of the M10 supercapacitor device was ~29.44 Ω at lower frequencies (1 kHz) and almost similar ~31.25 Ω at higher frequencies (50 kHz), as shown in the Nyquist plot (Figure S4d). The plot displayed a semicircle feature at the high-frequency end, and the vertical line represented a combination of Warburg resistance, leakage resistance, and mass capacitance. The behavior of the supercapacitor closely resembled that of an ideal capacitor, with the vertical lines nearly parallel to the imaginary axis, indicating excellent performance.[1] The electrochemical stability test of the supercapacitor at a current density of 1 mA cm^{-2} demonstrate excellent cyclic stability, as the device maintained its initial capacitance even after 1,000 cycles, Figure S4e. The inset in the figure illustrates the CV profile of the supercapacitor up to 1000 cycles, exhibiting no deviations. Figure S4f displays the charge-discharge curves from the 990th to the 1000th cycle of the GCD tests. The M10 supercapacitor, without the use of any current collector, achieved an areal energy density of 3.944 µWh cm^{-2} and a power density of 3550 µW cm^{-2}. Furthermore, it delivered a specific energy density of 1.578 Wh kg^{-1} and power density of 1420 W kg^{-1}.

Supporting information 6.

Comparison of capacitance performance of the heterostructure supercapacitors

Table S2. Overview of capacitance comparison of some reported supercapacitors in the literature

**Electrode**	**Electrolyte**	**Areal capacitance**	**Energy and Power density**	**Ref**
Graphene	PVA-H_2SO_4	3.2 mF cm^{-2}	0.28 mWh cm^{-2} and 3 mW cm^{-2}	[2]
NiO/MoS2/rGO	1 M KCl	7.38 mF cm^{-2} (at 25 mV s^{-1})		[3]
rGO-GO-rGO	0.1 M $NaClO_4$	2 mF cm^{-2}	2–5.4×10^{-4} Wh cm^{-2} and 3.6–9×10^{-2} W cm^{-2}	[4]
Light scribed GO	PVA-H_2SO_4	2.9 mF cm^{-2} (at 50 mV s^{-1})		[5]
rGO-CNT	3 M KCl	6.1 mF cm^{-2} (at 10 mV s^{-1})	0.68 mWh cm^{-3} and 77 W cm^{-3}	[6]
CNT/MoO3	1 M HCl	4.86 mF cm^{-2} (at 10 mV s^{-1})	2.70 μWh cm^{-2} and 0.53 mW cm^{-2}	[7]
Graphite oxide	1 M Na_2SO_4	0.51 mF cm^{-2} (at 20 mV s^{-1})		[8]
MoS_2 nanoparticles	0.5 M H_2SO_4	29 μF cm^{-2} (at 0.5 mV s^{-1})		[9]
Graphene/CNT	1 M Na_2SO_4	2.16 mF cm^{-2} (at 100 mA cm^{-2})	2.42 mWh cm^{-3} and 115 W cm^{-3}	[10]
Exfoliated MoS_2	6 M KOH	2 mF cm^{-2} (at 10 mV s^{-1})		[11]
Graphene/textile	PVA-H_2SO_4	80.185 mF cm^{-2} (at 1 mV s^{-1})	44.547 μWh cm^{-2} and 581.05 μW cm^{-2}	This study
MoS_2/textile	PVA-H_2SO_4	7.1 mF cm^{-2} (at 1 mV s^{-1})	3.944 μWh cm^{-2} and 3550 μW cm^{-2}	This study
MoS_2-graphene bi-layer/textile	PVA-H_2SO_4	63.73 mF cm^{-2} (at 1 mV s^{-1})	35.405 μWh cm^{-2} and 8497.333 μW cm^{-2}	This study
Graphene-MoS_2-graphene tri-layer/textile	PVA-H_2SO_4	105.08 mF cm^{-2} (at 1 mV s^{-1})	58.377 μWh cm^{-2} and 1604.274 μW cm^{-2}	This study

Reference

1. Potphode, D. D.; Sivaraman, P.; Mishra, S. P.; Patri, M. Polyaniline/Partially Exfoliated Multi-Walled Carbon Nanotubes Based Nanocomposites for Supercapacitors. *Electrochim. Acta* **2015**, *155*, 402-410, DOI: 10.1016/j.electacta.2014.12.126
2. Islam, M. R.; Afroj, S.; Beach, C.; Islam, M. H.; Parraman, C.; Abdelkader, A.; Casson, A. J.; Novoselov, K. S.; Karim, N. Fully Printed and Multifunctional Graphene-Based Wearable E-Textiles for Personalized Healthcare Applications. *iScience* **2022**, *25*, 103945, DOI: 10.1016/j.isci.2022.103945.
3. Ghasemi, F.; Jalali, M.; Abdollahi, A.; Mohammadi, S.; Sanaee, Z.; Mohajerzadeh, S. A High Performance Supercapacitor Based on Decoration of MoS_2/Reduced Graphene Oxide With NiO Nanoparticles. *RSC Adv.* **2017**, *7*, 52772-52781, DOI: 10.1039/C7RA09060A
4. Hu, Y.; Cheng, H.; Zhao, F.; Chen, N.; Jiang, L.; Feng, Z.; Qu, L. All-In-One Graphene Fiber Supercapacitor. *Nanoscale* **2014**, *6*, 6448-6451, DOI: 10.1039/C4NR01220H
5. Cai, F.; Tao, C.-a.; Li, Y.; Yin, W.; Wang, X.; Wang, J. Effects of Amount of Graphene Oxide and The Times of LightScribe on The performance of All-Solid-State Flexible Graphene-Based Micro-Supercapacitors. *Mater. Res. Express* **2017**, *4*, 036304, DOI: 10.1088/2053-1591/aa65fb
6. Beidaghi, M.; Wang, C. Micro-Supercapacitors Based on Interdigital Electrodes of Reduced Graphene Oxide and Carbon Nanotube Composites with Ultrahigh Power Handling Performance. *Adv. Funct. Mater.* **2012**, *22*, 4501-4510, DOI: 10.1002/adfm.201201292
7. Noh, J.; Yoon, C.-M.; Kim, Y. K.; Jang, J. High performance asymmetric supercapacitor twisted from carbon fiber/MnO_2 and carbon fiber/MoO_3. *Carbon* **2017**, *116*, 470-478. DOI:10.1016/j.carbon.2017.02.033
8. Gao, W.; Singh, N.; Song, L.; Liu, Z.; Reddy, A. L. M.; Ci, L.; Vajtai, R.; Zhang, Q.; Wei, B.; Ajayan, P. M. Direct Laser Writing of Micro-Supercapacitors on Hydrated Graphite Oxide Films. *Nat. Nanotechnol.* **2011**, *6*, 496-500, DOI: 10.1038/nnano.2011.110
9. Wang, H.; Lu, Z.; Kong, D.; Sun, J.; Hymel, T. M.; Cui, Y. Electrochemical Tuning of MoS_2 Nanoparticles on Three-Dimensional Substrate for Efficient Hydrogen Evolution. *ACS Nano* **2014**, *8*, 4940-4947, DOI: 10.1021/nn500959v
10. Lin, J.; Zhang, C.; Yan, Z.; Zhu, Y.; Peng, Z.; Hauge, R. H.; Natelson, D.; Tour, J. M. 3-Dimensional Graphene Carbon Nanotube Carpet-Based Microsupercapacitors With High Electrochemical Performance. *Nano Lett.* **2013**, *13*, 72-78, DOI: 10.1021/nl3034976
11. Winchester, A.; Ghosh, S.; Feng, S.; Elias, A. L.; Mallouk, T.; Terrones, M.; Talapatra, S. Electrochemical Characterization of Liquid Phase Exfoliated Two-Dimensional Layers of Molybdenum Disulfide. *ACS Appl. Mater. Interfaces* **2014**, *6*, 2125-2130, DOI: 10.1021/am4051316
